# An efficient low‐cost xylem sap isolation method for bacterial wilt assays in tomato

**DOI:** 10.1002/aps3.11335

**Published:** 2020-04-19

**Authors:** Bendangchuchang Longchar, Tarinee Phukan, Sarita Yadav, Muthappa Senthil‐Kumar

**Affiliations:** ^1^ National Institute of Plant Genome Research, Aruna Asaf Ali Marg New Delhi 110067 India

**Keywords:** negative pressure, *Ralstonia solanacearum*, tomato stem, xylem sap extraction

## Abstract

**Premise:**

A portable, simple, yet efficient method was developed for the rapid extraction of xylem sap from the stems and petioles of tomato plants for diagnostic and quantification assays of the xylem‐colonizing wilt bacterium *Ralstonia solanacearum*.

**Methods and Results:**

Xylem saps were extracted from tomato stem sections using negative pressure generated from handheld needleless syringes. The samples were collected from plants grown under different soil moisture levels at four days after inoculation with the pathogen. Pipette tips were modified to serve as adapters for the stem sections. The quantification of the bacterial load in the extracted sap was performed by plating sap dilutions in Kelman's triphenyltetrazolium chloride (TTC) medium. Pathogen identity was further confirmed by performing a PCR using *R*. *solanacearum*‐specific primers.

**Conclusions:**

Due to its simplicity, portability, and thoroughness of extraction from predetermined tissue sizes, the method can potentially facilitate high‐throughput onsite sampling from a large number of samples in a short time, which cannot be achieved with other available techniques.

*Ralstonia solanacearum* is a soil‐borne β‐proteobacterium that causes bacterial wilt disease in over 200 plant species belonging to more than 50 different families (Hayward, 1991; Elphinstone, 2005). *Ralstonia solanacearum* is a xylem‐colonizing pathogen that invades the host xylem tissues through natural openings or wounds in the roots (Tans‐Kersten et al., 2001; Caldwell et al., 2017). Once inside, the pathogen spreads systemically throughout the host xylem network, growing into colony aggregates in a biofilm matrix that may clog and disrupt water flow in the vessels (Minh Tran et al., 2016). The pathogen moves within the infected plant as planktonic cells in the xylem sap flow or through twitching motility along the vessel walls (Liu et al., 2001). *Ralstonia solanacearum* turns virulent after attaining a certain population density (>10^8^ CFU/g stem or >10^9^ CFU/mL xylem sap), releasing virulence factors, such as exopolysaccharides, cell‐wall degrading enzymes, and type III effectors, via a complex regulatory system (Genin and Denny, 2012).

Although susceptible tomato (*Solanum lycopersicum* L.) genotypes typically wilt at such bacterial concentrations, several resistant genotypes are known to tolerate concentrations up to 10^7^ CFU/g stem without wilting symptoms (Grimault and Prior, 1993). Tomato resistance against *R*. *solanacearum* is based on tolerance, which is influenced by factors such as changes in temperature, humidity, and pathogen density (Hayward, 1991); thus, changing the planting time and growing conditions could help enhance plant tolerance to the wilt disease. Owing to its economic importance, several control methods for bacterial wilt have been developed over the years and are currently employed in risk areas to mitigate crop loss (Yuliar et al., 2015). However, implementing any successful control measure would crucially depend on the timely and proper identification of the pathogen from affected fields, requiring the collection of pathogen samples from plant tissues, such as stems, roots, or petioles, for accurate quantification and diagnostic analyses in laboratories.

The collection of xylem sap from infected tomato plants is an important step in estimating the bacterial load within the plant at different stages of infection during pathogenicity and plant resistance assays. An interesting feature of *R*. *solanacearum* is its tendency to frequently become avirulent while remaining capable of colonizing plants (Kelman and Hruschka, 1973; Brumbley and Denny, 1990; Peyraud et al., 2016; Zheng et al., 2017). Avirulent strains of *R*. *solanacearum* have been effectively used as a biological control agent against the wilt pathogen. This biocontrol property is likely effected through a competition for resources as avirulent strains have a higher growth and metabolic rate than the virulent strains (Yuliar et al., 2015; Peyraud et al., 2016). Analyzing extracted xylem sap could potentially help identify the pathogen strains and their potential threat or benefit to field crops. The rapid extraction of xylem fluids from determinate plant tissues is also crucial for final diagnostics and the quantification of other xylem‐colonizing plant pathogens. An efficient extraction technique is therefore important for the sensitive detection of the pathogen and also for host–pathogen interaction studies (Bell et al., 1995).

One of the simplest approaches for collecting xylem exudates is to make a cross‐section excision in the stem and harvest the sap oozing out through capillary movement and root pressure (Buhtz et al., 2004; Rellán‐Álvarez et al., 2011; Lowe‐Power et al., 2018). The gravitated bacterial ooze containing heavy‐molecular‐weight exopolysaccharides streaming from cut upper aerial stems of symptomatic plants can also be collected for diagnostic use (Kinyua et al., 2014). However, the use of these approaches for high‐throughput and sensitive assays can be restricted by limitations, including being (a) time consuming, because bacterial (xylem sap) oozing through either the ascent of sap or gravity is a slow process; and (b) inconsistent and inaccurate, because the size of tissues are variable, the rate of oozing varies between plants, and the bacterial population dynamics (doubling time of 4 h) change during the variable waiting periods. Additionally, sap oozing is virtually absent from plants under moisture‐deficit stress, making sampling through the above approaches unfeasible. Several studies involving xylem sap extraction have reported using the Scholander pressure bomb, an instrument that is primarily designed for measuring water potentials of tissue samples (Scholander et al., 1965; Bextine and Miller, 2004; Netting et al., 2012; Flajšman et al., 2017). Although effective, the Scholander pressure instrument is expensive, and may not be feasible for large‐scale sampling in limited time periods.

In this study, we describe a simple yet efficient xylem extraction technique that allows rapid sampling from large numbers of plants grown under both optimal and moisture‐deficit stress conditions. The extraction unit is highly portable and can be easily set up in the lab or field to begin immediate onsite sampling (sap extraction), which is not possible with current techniques. It allows for convenient in‐field sample collection and handling in microfuge tubes, rather than transporting entire plants. This ability for high‐throughput sampling within short periods of time is crucial considering the multiplication cycle of the pathogen, and thus enables the accurate determination of pathogen densities. Analyses of xylem saps collected from petioles could potentially help identify infected plants and the stage of infection well before the onset of the wilting disease in affected fields.

## METHODS AND RESULTS

### Plant growth, pathogen, and moisture stress imposition

The tomato (*Solanum lycopersicum*) variety Pusa Ruby, a genotype susceptible to bacterial wilt procured from the Indian Agricultural Research Institute (IARI), New Delhi, was used in this study. The seeds were sown into a sterile soil mix (agropeat and perlite in a 3 : 1 ratio), then maintained in a growth room at a temperature of 25 ± 2°C, a humidity of 60%, and in a 12‐h/12‐h photoperiod. Individual seedlings were transferred to 7.6 × 7.6‐cm pots 10 days after germination and grown in the growth room. To study the volume of xylem sap and the bacterial load therein under different soil moisture levels, two‐week‐old plants were maintained at 100%, 80%, 60%, 40%, and 20% field capacities (FC) by withholding irrigation to pots using the gravimetric method (Ramegowda et al., 2013).

The bacterial pathogen *R*. *solanacearum* strain F1C1 (Kumar et al., 2013), tagged with the *mCHERRY* marker gene (TRS1016) (Monteiro et al., 2012; Capela et al., 2017; Singh et al., 2018), was used as the inoculum for the pathogenicity assays. Glycerol stocks of the pathogen were streaked on Kelman's (CPG; Kelman, 1954) agar media (1.0% bacteriological peptone [P], 0.1% yeast extract, 0.1% casamino acid [C], and 0.5% glucose [G]) containing 0.005% triphenyltetrazolium chloride (TTC). Single colonies were used for culturing bacterial suspensions in CPG broth media. To select the TRS1016‐carrying *R*. *solanacearum* strain, gentamycin (50 μg/mL) was used. For the pathogen inoculation, bacterial suspensions were prepared in sterile water and adjusted to OD_600_ = 0.1 (1 × 10^8^ CFU/mL). Before inoculation, the roots of the tomato plants were uniformly injured by inserting a scalpel blade into the soil around the stem. Thereafter, 5 mL of the inoculum was poured into the soil approximately 2.5 cm away from the stem. For the control, the roots of the plants were similarly injured as above and 5 mL of sterile water was used as the mock inoculum. Wilting symptoms started appearing at four days post‐inoculation (DPI) with the pathogen in the well‐irrigated plants (100% and 80% FC). Tissues were sampled from all the plants for xylem sap extraction immediately upon the appearance of bacterial wilt symptoms in the pathogen‐inoculated non‐moisture‐stressed plants.

### Xylem sap extraction unit

**Figure 1:**
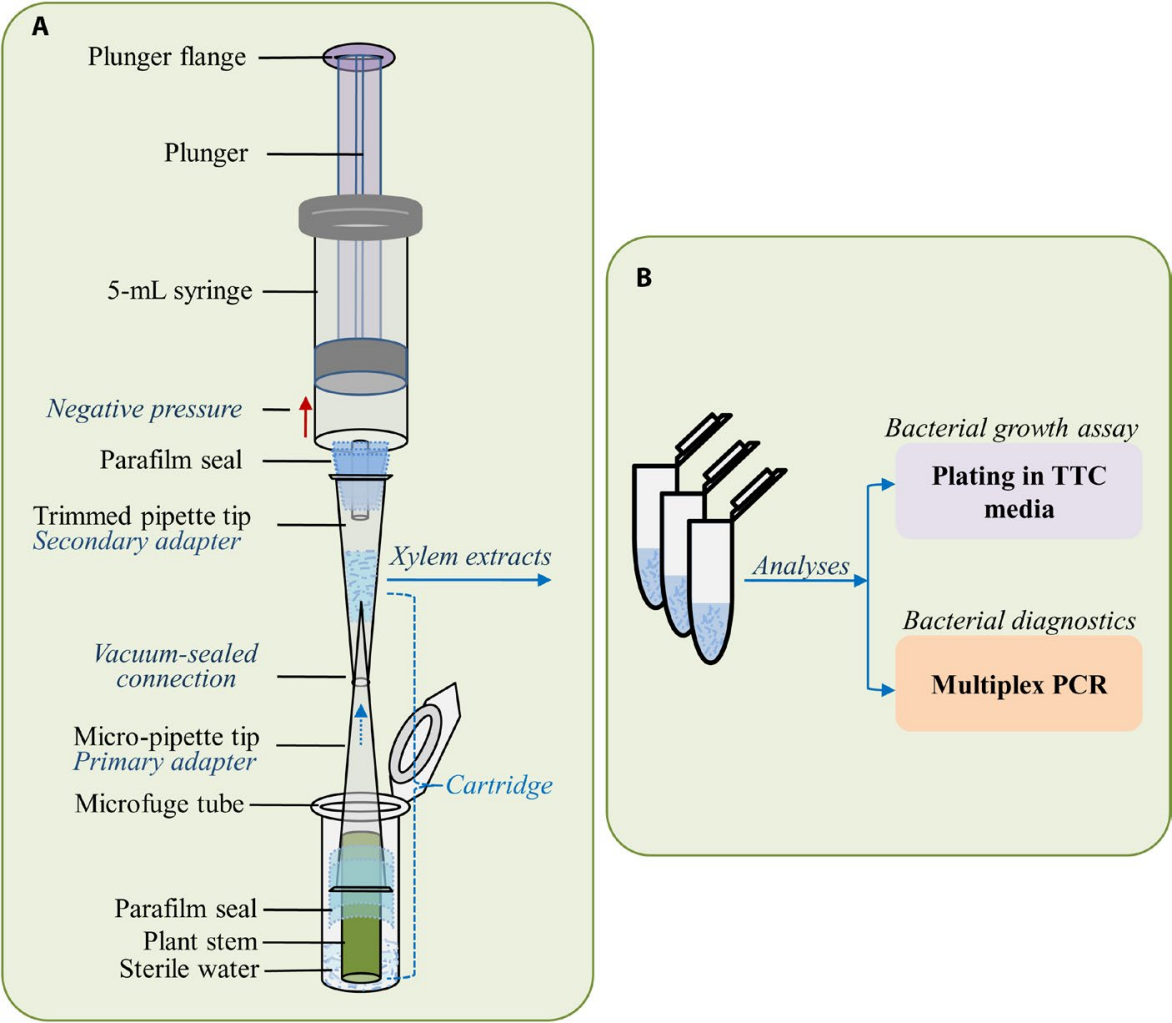
Illustration of the portable negative pressure xylem sap extraction unit and downstream analytical steps. (A) A fully assembled unit consisting of a pressure‐generating needleless syringe with nozzle diameter resized using a parafilm strip, a secondary adapter made of a trimmed micropipette tip for collecting and dispensing the extracted saps, “cartridges” composed of the plant stem section fitted into a micropipette tip and sealed with thin parafilm strips, and a microfuge tube containing the desired amount of sterile water. (B) The extracted sap can be used for downstream diagnostic and quantification assays for *Ralstonia solanacearum*, including plating in triphenyltetrazolium chloride (TTC)–containing media and multiplex PCR for pathogen identification.

**Figure 2:**
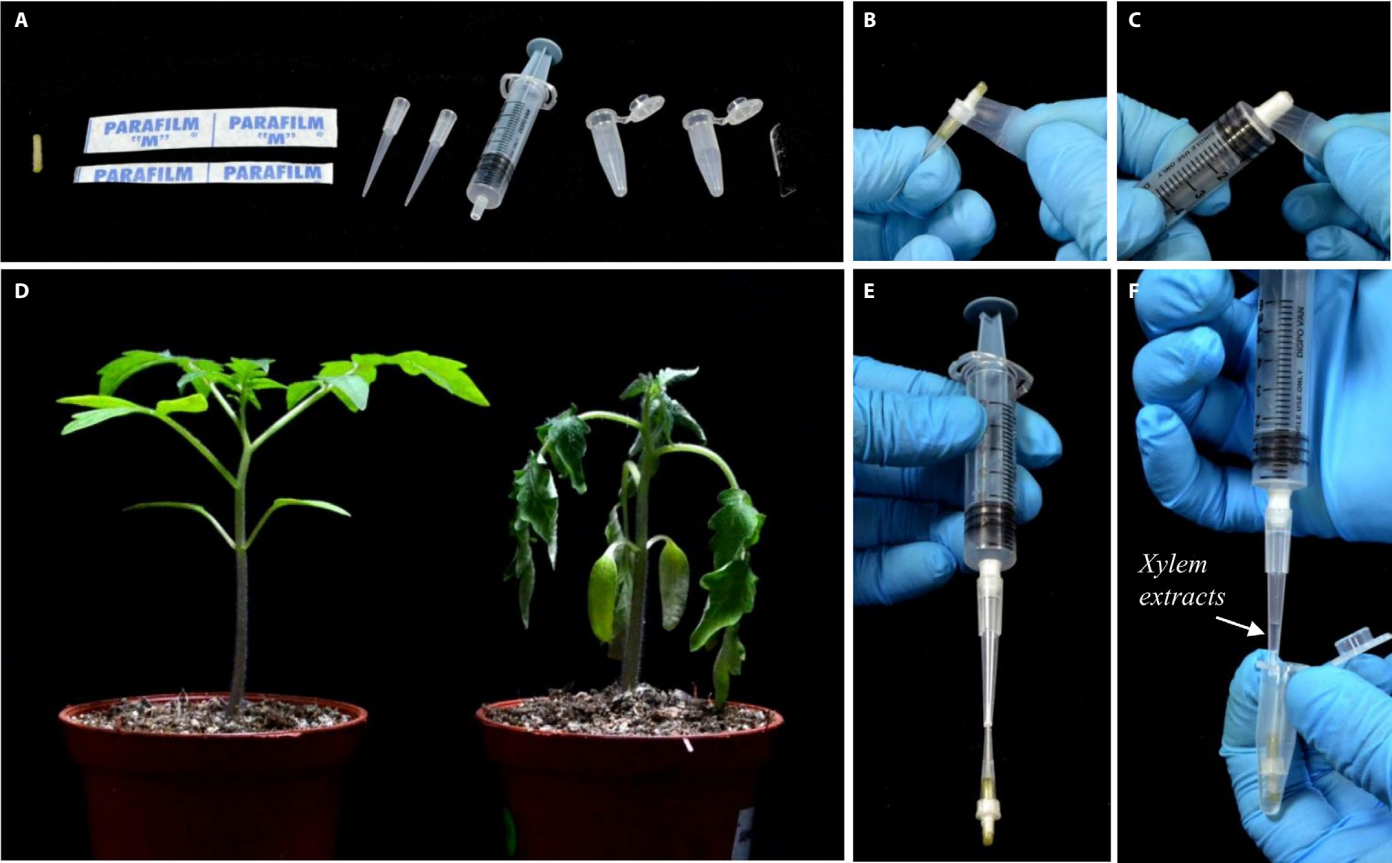
Xylem sap extraction from tomato stems using negative pressure. (A) The essential components needed for constructing the negative pressure extraction unit, including a 2‐cm stem sample, parafilm strips, micropipette tips of desired capacities, a needleless syringe, microcentrifuge tubes, and a scalpel. (B) Fabrication of a cartridge (stem sample fitted onto a micropipette tip and sealed with parafilm). (C) Resizing of the syringe nozzle with a parafilm strip for fitting the secondary adapters. (D) Tomato plant showing typical bacterial wilt disease symptoms (right) at four days post‐inoculation with *Ralstonia solanacearum* compared with a healthy uninoculated plant (left). (E) The completed set up of the negative pressure extraction unit. (F) Xylem sap extracted from one of the samples using 100 μL of sterile water in a microfuge tube.

A sterile needleless syringe (preferably 1‐mL and 5‐mL capacities) was used to generate negative pressure in the plant tissues (Figs. 1, 2). The nozzle‐adapter of the syringe was resized by winding a parafilm strip (~30–40 mm wide) around the nozzle to fit a micropipette tip (200‐μL capacity) (Figs. 1A, 2C). A secondary adapter fitting this modified nozzle was made by transversely cutting off ~30 mm from the pointed end of a 200‐μL micropipette tip using a sharp, sterile scalpel blade (Fig. 1A). Cartridges were made using a 10‐μL micropipette tip (primary adapter) with the tissue fixed firmly into the tip; the tip–tissue junction was sealed with a parafilm strip (Fig. 2B). For the plant tissues, 2‐cm lengths of stems were harvested from below the cotyledonary node. The tip of the primary adapter of the cartridge was then firmly attached into the trimmed end of the secondary adapter. Thereafter, the secondary adapter was firmly attached onto the modified syringe nozzle, ensuring an air‐tight connection (Figs. 1A, 2E).

The xylem extraction was performed by gently applying negative pressure to the plant stem, the exposed distal end of which was dipped in ~100 μL of sterile water in a sterile 1.5‐mL microfuge tube (Fig. 2F). The cartridge was then removed from the water after approximately 50 μL of sap was collected in the secondary adapter, but the pressure application was continued. At this stage, the sap content in the xylem conduits would have collected as the water was sucked in through the distal end of the stem. After a few seconds of continued suction, bubbles (froth) started to appear from the proximal end of the stem, which eventually ceased, indicating a complete extraction. The pressure in the syringe was released by simply letting go of the thumb from the plunger flange (piston). Once the pressure in the barrel chamber was released (i.e., the released plunger came to a standstill), the cartridge was slowly and gently detached from the secondary adapter, and the harvested sap was dispensed into the microfuge tube and mixed with the remaining water. It was important to release the pressure before removing the cartridge from the secondary adapter to avoid the entire sap sample being sucked into the barrel chamber.

In order to accurately determine the exact volume of xylem sap from a determinate tissue length, the collection of the entire fluid was essential. To achieve this, the spent cartridges were placed into corresponding tubes and briefly spun in a centrifuge to collect any trace of trapped fluids inside the cartridge. When large numbers of samples are involved, this step need not be performed for all samples as it would be time consuming. Instead, the mean value from a minimum of four samples can be taken to account for a given experiment in which uniform tissue samples were used. The volume of actual xylem sap was determined by subtracting the volume of sterile water used from the mean total extraction sample volume; however, we found that even after spin‐harvesting the sap, a small volume of extraction solution was invariably lost. Using spent cartridges to imbibe 100 μL of sterile water, we determined that approximately 10 ± 2 μL of volume was lost; thus, the final actual xylem sap volume was calculated by subtracting 90 μL of water from the mean volume of total sap extract.

A detailed protocol for the xylem sap extraction is provided in Appendix 1.

### In planta bacterial growth assay

The extracted xylem saps were serially diluted using sterile water, and 10 μL of each dilution was plated on CPG agar medium supplemented with TTC and gentamycin. The plates were incubated at 28°C for 48 h, after which the colonies were counted. The bacterial count was assayed from four biological and two technical replicates for each treatment. The following formula was used to calculate the colony‐forming units (CFU):

$${\text{Log}}_{10}\;\text{CFU/mL}=\frac{(\text{No. of colonies}\times \text{volume of suspension}\times \text{dilution factor)}\times 1000}{\text{Volume of suspension plated}\times \;\text{volume of xylem sap}}$$

**Figure 3:**
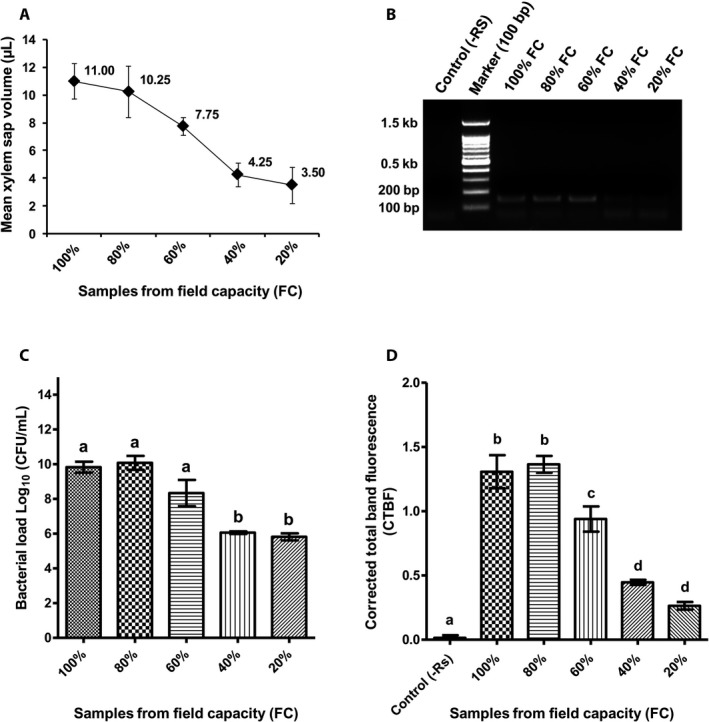
Determination and quantification of *Ralstonia solanacearum* from xylem sap extract. (A) The mean volume of xylem sap in 2‐cm stem tissues of *R. solanacearum*–inoculated tomato plants grown at different soil moisture levels: 100%, 80%, 60%, 40%, and 20% field capacities (FCs). Each mean value was derived from four biological replicates. (B) Agarose gel image of a phylotype‐specific multiplex PCR showing the amplification of the 144‐bp ITS fragment specific to the Phylotype I group of *R*. *solanacearum* used in this study. The amplifications confirmed an *R*. *solanacearum* infection in all the inoculated plants from different treatments (% FCs), but not in the control (water‐inoculated) plants. The image shows the PCR amplification of only one representative biological sample from each treatment. (C) Graph showing the bacterial load in the xylem saps of *R. solanacearum–*inoculated tomato plants grown under different soil moisture levels (% FCs). The bacterial growth curve was generated using the colony count method and is represented as the log_10_ CFU/mL. (D) PCR‐based quantification of *R. solanacearum* load in xylem sap. The graph displays the fluorescence intensities generated from agarose gel images of the PCR‐amplified ITS fragment of *R. solanacearum* isolated from the xylem saps of infected tomato plants grown at different soil moisture levels. Xylem extracts from water‐inoculated plants were used as the control. For each treatment (% FC), four biological replicates (plants) were used. Quantification values were generated using ImageJ version 1.48. Different letters in parts C and D indicate statistically significant differences (*P* < 0.05; one‐way ANOVA with Tukey's multiple comparison test.

Because the total volume of xylem saps extracted from plants grown at different soil moisture levels (% FC) were different, we first determined the mean volume of total sap extract and the mean volume of actual xylem sap content for each FC treatment. The mean volumes of actual xylem sap in the total sap suspension from 100%, 80%, 60%, 40%, and 20% FCs were determined as 11.00, 10.25, 7.75, 4.25, and 3.50 μL per 2‐cm stem, respectively (Fig. 3A). The mean bacterial loads (density) in the sap samples from the plants grown at 100%, 80%, and 60% FC were determined to be 2.0 × 10^10^, 7.2 × 10^10^, and 2.9 × 10^10^ CFU/mL, respectively, which was consistent with the established load required for wilt disease to manifest (Genin and Denny, 2012). The logarithmic value of the bacterial load in xylem saps extracted from the plants grown at 100%, 80%, and 60% FC were statistically not significant (Fig. 3C); however, the pathogen load was significantly lower in moisture deficit–stressed plants grown at 40% and 20% FC (1.1 ×10^6^ and 8.0 × 10^5^ CFU/mL, respectively), which was correlated with their lower xylem sap content (Fig. 3A, C). This translated to a delayed and reduced frequency of wilting under the moisture deficit–stressed conditions.

### Multiplex PCR

In order to genetically identify *R*. *solanacearum* and differentiate between different phylotypes, a multiplex PCR method was used, as described by Fegan and Prior (2005). The set of five unique primers used for the phylotype‐specific multiplex PCR are based on the DNA sequences of the internal transcribed spacer (ITS) region in the *R*. *solanacearum* genome (Table 1) (Fegan and Prior, 2005). The 20‐μL PCR reaction for each sample contained 2.0 μL of 10× Optimized DyNAzyme Buffer (Thermo Fisher Scientific, Waltham, Massachusetts, USA), 1.0 μL of 50 mM MgCl_2_, 0.5 μL of 10 mM dNTP mix (Promega Corporation, Madison, Wisconsin, USA), 1.0 μL each of 10 mM primer (Eurofins Scientific, Luxembourg City, Luxembourg), 0.2 μL of DyNAzyme II DNA Polymerase (Thermo Fisher Scientific), 1.0 μL of template DNA from the xylem extract (see below), with the final volume adjusted to 20 μL with nuclease‐free H_2_O (HiMedia Laboratories, Mumbai, India). The template DNA was prepared as described by Kumar et al. (2013), with minor modifications: into 9.5 μL of xylem extract, 0.5 μL of 200 mM NaOH was added and the solution was incubated at 95°C for 10 min, after which 1.0 μL was used in the PCR reaction. The reaction was run in a thermal cycler (C1000 Touch Thermal Cycler, Bio‐Rad Laboratories, Hercules, California, USA) with the following PCR conditions: 96°C for 5 min; followed by 29 cycles of 94°C for 20 s, 59°C for 30 s, and 72°C for 45 s; with a final extension of 72°C for 10 min. For agarose gel electrophoresis, 3.0 μL of each PCR product was loaded onto and resolved in a 1.2% agarose gel containing 0.2 μg/mL ethidium bromide, then imaged in a Universal Hood II Gel Doc System (Bio‐Rad Laboratories). The PCR amplified a 144‐bp ITS fragment specific to the Phylotype I group of *R*. *solanacearum*, which was consistent with the phylotype to which the mCHERRY‐F1C1 strain (TRS1016) used in this study belonged (Fig. 3B). In addition to the identification of the pathogen, the PCR amplifications also confirmed the presence of *R*. *solanacearum* infection in all inoculated plants from the different treatments (FCs).

**Table 1:** List of primers targeting the ITS region used for the phylotype‐specific multiplex PCR (Fegan and Prior, 2005).

Primer name	Primer sequences (5′–3′)	Length (nucleotides)	Phylotype specificity	Amplicon size (in bp) with Nmult22:RR
Nmult21:1F	F: CGTTGATGAGGCGCGCAATTT	21	Phylotype I	144
Nmult21:2F	F: AAGTTATGGACGGTGGAAGTC	21	Phylotype II	372
Nmult23:AF	F: ATTACSAGAGCAATCGAAAGATT	23	Phylotype III	91
Nmult22:InF	F: TTGCCAAGACGAGAGAAGTA	20	Phylotype IV	213
Nmult22:RR	R: TCGCTTGACCCTATAACGAGTA	22	All phylotypes	NA

Note

NA = not applicable.

### Semi‐quantitative PCR analysis of bacterial load in xylem sap

In order to perform a rapid comparative analysis of xylem sap bacterial density, we used a PCR‐based fluorescence quantification assay that was compared with the standard in planta bacterial growth curve. For this, the gel fluorescence intensities of a PCR‐amplified *R*. *solanacearum* ITS fragment from the xylem saps of tomato plants grown at different soil moisture levels were used. Xylem extracts from uninoculated plants were used as a negative control. For each treatment (% FC), four biological replicates were performed. The template preparation and PCR conditions were as described above, and 2 μL of PCR product was loaded in a 1.2% agarose gel containing 0.2 μg/mL of ethidium bromide. Fluorescence values were generated using ImageJ software (version 1.48; Abramoff et al., 2004). The set measurements included the area, integrated density, and mean gray value, selected from the Analyze menu in ImageJ. The amplification fluorescence intensity was normalized using at least 10 readings from background gel areas with no fluorescence. The control values were taken from the same region where an amplification of a 144‐bp fragment would be expected to run. The area selected for all points was 533.4 μM^2^. The corrected total band fluorescence (CTBF) was calculated using the following formula:

CTBF=integrated density-(area of selected cell×mean background fluorescence)

The graph generated from the plotted CTBF values showed that pathogen infection occurred at all soil moisture levels (Fig. 3D); however, there were significant differences in the fluorescence intensities between different treatments, suggestive of differences in bacterial load. This is shown by the similarity between the fluorescence graph and the in planta bacterial load curve generated from the corresponding xylem sap extracts of a replicated experiment (Fig. 3C). The significant fluorescence intensity differences between plants with soil moisture levels of 100/80/60% FC and 40/20% FC were also consistent with the pathogen load and mean volumes of xylem extract from the corresponding treatments (Fig. 3A, C). The PCR‐based assay is a convenient way to rapidly determine pathogen identities and relative quantities in different plant samples. We could not consistently obtain adequate xylem sap through root pressure exudation from plants at 40% FC, and never achieved this at 20% FC; however, using the negative‐pressure isolation method, we were able to both harvest the xylem sap from such samples as well as confirm the presence of *R*. *solanacearum* using multiplex PCR.

## CONCLUSIONS

The accurate and rapid detection of plant pathogens is very important, particularly for pathogens causing diseases in crop plants. *Ralstonia solanacearum* is ranked among the top 10 economically important plant pathogens and has a broad host range, many of which are popular crop plants. Determining *R*. *solanacearum* from other wilt‐disease‐causing plant pathogens is crucial for the implementation of proper control measures. Wilt and other disease symptoms can also be caused by other pathogens, such as *Fusarium* spp., *Verticillium* spp., *Dickeya dadantii*, and *Clavibacter michiganensis*, which may be potentially confused with symptoms caused by *R*. *solanacearum* (OEPP/EPPO, 2004). Although an onsite diagnosis can be made by conventionally observing bacterial ooze streaming in water or ooze drops in cut stems, or by using commercially available immunostrips, an accurate and definitive diagnosis of *R*. *solanacearum* can only be achieved through laboratory analyses.

One crucial step for the large‐scale screening of field crop plants for a xylem‐colonizing pathogen, such as *R*. *solanacearum*, is to have an efficient and portable method for sampling the xylem saps. The availability of xylem saps can facilitate the use of sensitive tests to identify the pathogen and determine the degree of infection. In contrast to the currently available methods for xylem sap collection (Scholander et al., 1965; Buhtz et al., 2004), our protocol offers an efficient, rapid, and high‐throughput sap extraction method with a high level of accuracy. The advantages of the new method over other extraction techniques are underscored by its (a) *simplicity*, as it requires cheap and easily available components to set up a fully functional unit and demands only minimal skills for handling; (b) *portability and versatility*, as it is small, does not require any external source of power, and can be modified according to need; (c) *precision*, as pre‐determined uniform tissue sample sizes can be used, thus allowing the accurate determination of the xylem sap content; (d) *speed and up‐scalability*, as the cartridges can be quickly prefabricated and the extraction can be performed within seconds for each sample, thus enabling rapid extraction from large numbers of samples; and (e) *sensitivity and efficacy*, as it can be effectively used even in plants growing under drought stress with much lower sap contents.

Our new method is especially ideal for extraction from uniformly structured tissues, such as stem and petiole sections. It may, however, not be best suited for sap extraction from root and leaf tissues, for which further modifications of the adapters may be required. Additionally, the presence of trace amounts of phloem sap in the extracts cannot be ruled out because both the phloem and xylem tissues in soft herbaceous plants, such as tomato, are enclosed within the vascular bundle as a single unit. Clear differences in the anatomy and physiological functions of these two tissues indicate that the extracts obtained using this method will predominantly be of xylem origin. Using this method, two researchers could extract the xylem sap from 40 different plant samples in less than 30 min. In contrast, collecting root pressure–generated sap exudates from the cut stems of each plant sample took highly variable amounts of time (30 min to 12 h) and yielded variable quantities of saps. A drawback associated with this variability is the effect on the pathogen population dynamics, for example, *R*. *solanacearum* normally has a doubling time of 4 h. It is important to note that no sap exudates were produced from the cut stems from plants grown in low soil moisture levels (20% and 40% FCs), and hence no sap could be sampled. With the new method, however, saps could be easily and rapidly extracted from all samples under all moisture stress levels. The development of this protocol was driven by our need to obtain xylem saps from moisture‐stressed plants in our combined stress biology investigations.

Our method offers researchers a low‐cost, simple, but very efficient extraction technique that can be used not only for plant pathogen detection, but also for studying the physio‐biochemical dynamics of xylem sap under different aspects of plant stress biology.

## AUTHOR CONTRIBUTIONS

M.S‐K. and B.L. conceived and designed the experiment. B.L., T.P., and S.Y. performed the experiments. B.L. and M.S‐K.drafted the manuscript. B.L., M.S‐K., T.P., and S.Y. critically reviewed, commented, and revised the manuscript. All the authors gave the approval for publication of the final revised version.

## Appendix 1 Protocol for constructing a handheld negative pressure xylem sap extraction unit.

### Materials required for xylem sap extraction unit

- Needleless syringes (1‐mL, 5‐mL, 10‐mL, etc. capacities; Dispo Van Hindustan Syringes and Medical Devices Ltd., Ballabhgarh, Faridabad, India)
- Parafilm strips (Bemis Company Inc., Neenah, Wisconsin, USA)
- Pipette tips (autoclavable graduated tips in 10‐μL, 200‐μL, and 1000‐μL capacities; Tarsons Products Pvt. Ltd., Shakespeare Sarani, Kolkota, India)
- Microfuge tubes (0.5 mL, 1.5 mL, and 2 mL; Tarsons Products Pvt. Ltd.)
- Sterile surgical scalpel blades (Hindustan Surgicals, Kanpur, India)

### Extraction procedure

1. Take a sterile needleless syringe (preferably 1‐mL or 5‐mL capacity) and resize the syringe nozzle adapter by winding around the nozzle with a parafilm strip (~30–40 mm width) to fit a micropipette tip (200‐μL capacity) (Figs. 1A, 2C). Approximately eight to 12 layers will sufficiently thicken the nozzle to snugly fit a 200‐μL micropipette tip (secondary adapter).
2. Assemble the secondary adapter: Using a sharp, sterile scalpel blade, transversely cut off ~30 mm from the pointed end of a 200‐μL micropipette tip. The required number of secondary adapters may be prepared according to the number of sample plant tissues to be collected.
3. Preparation of cartridges: Take a 10‐μL micropipette tip (primary adapter; choice of tip capacity can vary depending on the size of the tissues), excise a 2‐cm‐long stem/petiole (from below the cotyledonary node for the stem or above the attachment node for the petiole), fix the tissue gently but firmly into the micropipette tip, and seal using a parafilm strip (30–40 mm width). The parafilm strip must first be thinned by gently stretching and then wound around the tip and tissue, ensuring firm contact with the tissue at the midpoint of the exposed region (Fig. 2B). This step is crucial for creating an airtight seal at the junction of the tip and the tissue. It is crucial not to cover the distal end of the tissue, and at least 2–3 mm of plant tissue be should exposed. Care should be taken to not bend or injure the tissue, as this would disrupt the continuity of the xylem conduits and prevent xylem sap extraction.
4. Firmly attach the primary adapter tip of the cartridge onto the trimmed end of the secondary adapter (Figs. 1A, 2E).
5. Firmly fix the secondary adapter into the modified syringe nozzle, ensuring an airtight seal. Alternatively, the cartridge can be fixed after attachment of the secondary adapter into the nozzle (Figs. 1A, 2E). Before the attachment of the secondary adapter, the plunger may be pushed up slightly from a dispensed state to fill approximately 200 μL of air in the barrel. This step helps make the dispensing of the harvested sap much easier.
6. Dispense ~100 μL of sterile water into labeled sterile 1.5‐mL microcentrifuge tubes.
7. Place the exposed distal end of the plant tissue into the water, taking care to avoid the parafilm strip margin coming in contact with the water. This step will prevent any possibility of the water being aspirated through gaps between the tissue and the parafilm, bypassing the xylem tissues.
8. Once in contact with the water, aspirate by pushing up the plunger flange with the thumb to an approximate volume of 1 mL. Maintain the pressure on the flange until the sap starts accumulating in the tip of the secondary adapter (Fig. 2F).
9. Remove the tissue from the water after approximately 50 μL of sap has accumulated. Continue to maintain the negative pressure until bubbles start forming on the proximal end of the tissue sample. It is worth noting that the speed of extraction from infected tissues is significantly slower than from uninfected tissues; however, it is advisable to maintain a uniform gentle pressure throughout the extraction to avoid high‐pressure‐induced cell collapse.
10. Once the bubble formation has subsided, gently release the pressure by removing the thumb from the plunger flange and waiting for about 10 s or until the plunger stands still. This step is important as removing the cartridge from the adapter before releasing the pressure may lead to the entire sap sample being sucked into the syringe barrel.
11. Slowly remove the cartridge adapter tip from the secondary adapter and dispense the collected sap into the collection tube containing the remaining water. The sap suspension may be mixed by pipetting two to three times using the syringe to remove any trace of adhering sap on the adapter walls.
12. Determine actual sap content: For experiments with uniform tissue sizes, take at least 3–4 of the spent cartridges into the corresponding tube (primary adapter downwards) and spin briefly to collect any trace of trapped sap. Then, remove the tissue and dispense the trace collected sap in the adapter (if any) into the main suspension. The mean volume of these samples may be taken to calculate the actual mean sap volume per tissue by subtracting the volume of sterile water used. Because some volume of samples is invariably lost, first determine the mean volume lost by aspirating 100 μL of water using 3–4 spent cartridges. This *aspirated mean volume of water* (Y) can be subtracted from the *mean total extract* (EW) to obtain the *mean actual sap content* (X), calculated with the formula:
  X=EW-Y
13. Discard the spent cartridges and secondary adapters in suitable collection bags for proper disposal.
14. Tubes containing *R*. *solanacearum* in the harvested saps may be stored at 4°C from a few hours to several weeks, or for much longer durations at room temperature after further dilutions with sterile water. However, for certain downstream assays, such as bacterial load estimation and virulence tests, freshly extracted saps should be used to avoid potential cell growth in the sap suspension or cell death and loss of virulence due to prolonged storage.

## References

[aps311335-bib-0001] Abramoff, M. D. , P. J. Magalhaes , and S. J. Ram . 2004 Image processing with ImageJ. Biophotonics International 11(7): 36–42.

[aps311335-bib-0002] Bell, C. R. , G. A. Dickie , W. L. G. Harvey , and J. W. Y. F. Chan . 1995 Endophytic bacteria in grapevine. Canadian Journal of Microbiology 41: 46–53.

[aps311335-bib-0003] Bextine, B. R. , and T. A. Miller . 2004 Comparison of whole‐tissue and xylem fluid collection techniques to detect *Xylella fastidiosa* in grapevine and oleander. Plant Disease 88: 600–604. 30812578 10.1094/PDIS.2004.88.6.600

[aps311335-bib-0004] Brumbley, S. M. , and T. P. Denny . 1990 Cloning of wild‐type *Pseudomonas solanacearum phcA*, a gene that when mutated alters expression of multiple traits that contribute to virulence. Journal of Bacteriology 172: 5677–5685. 2211505 10.1128/jb.172.10.5677-5685.1990 PMC526882

[aps311335-bib-0005] Buhtz, A. , A. Kolasa , K. Arlt , C. Walz , and J. Kehr . 2004 Xylem sap protein composition is conserved among different plant species. Planta 219: 610–618. 15064951 10.1007/s00425-004-1259-9

[aps311335-bib-0006] Caldwell, D. , B. S. Kim , and A. S. Iyer‐Pascuzzi . 2017 *Ralstonia solanacearum* differentially colonizes roots of resistant and susceptible tomato plants. Phytopathology 107: 528–536. 28112595 10.1094/PHYTO-09-16-0353-R

[aps311335-bib-0007] Capela, D. , M. Marchetti , C. Clerissi , A. Perrier , D. Guetta , C. Gris , M. Valls , et al. 2017 Recruitment of a lineage‐specific virulence regulatory pathway promotes intracellular infection by a plant pathogen experimentally evolved into a legume symbiont. Molecular Biology and Evolution 34: 2503–2521. 28535261 10.1093/molbev/msx165

[aps311335-bib-0008] Elphinstone, J. G. 2005 The current bacterial wilt situation: A global overview *In* Allen C., Prior P., and Hayward A. C. [eds.], Bacterial wilt disease and the *Ralstonia solanacearum* species complex, 9–28. APS Press, St. Paul, Minnesota, USA.

[aps311335-bib-0009] Fegan, M. , and P. Prior . 2005 How complex is the *Ralstonia solanacearum* species complex *In* Allen C., Prior P., and Hayward A. C. [eds.], Bacterial wilt disease and the *Ralstonia solanacearum* species complex, 449–461. APS Press, St. Paul, Minnesota, USA.

[aps311335-bib-0010] Flajšman, M. , S. Mandelc , S. Radišek , and B. Javornik . 2017 Xylem sap extraction method from hop plants. Bio‐Protocol 7: e2172. 10.21769/BioProtoc.2172 PMC8376539 34458483

[aps311335-bib-0011] Genin, S. , and T. P. Denny . 2012 Pathogenomics of the *Ralstonia solanacearum* species complex. Annual Review of Phytopathology 50: 67–89. 10.1146/annurev-phyto-081211-173000 22559068

[aps311335-bib-0012] Grimault, V. , and P. Prior . 1993 Bacterial wilt resistance in tomato associated with tolerance of vascular tissues to *Pseudomonas solanacearum* . Plant Pathology 42: 589–594.

[aps311335-bib-0013] Hayward, A. C. 1991 Biology and epidemiology of bacterial wilt caused by *Pseudomonas solanacearum* . Annual Review of Phytopathology 29: 65–87. 10.1146/annurev.py.29.090191.000433 18479193

[aps311335-bib-0014] Kelman, A. 1954 The relationship of pathogenicity of *Pseudomonas solanacearum* to colony appearance in a tetrazolium medium. Phytopathology 44: 693–695. 10.1094/Phyto-44-693 40934411

[aps311335-bib-0015] Kelman, A. , and J. Hruschka . 1973 The role of motility and aerotaxis in the selective increase of avirulent bacteria in still broth cultures of *Pseudomonas solanacearum* . Journal of General Microbioogy 76: 177–188. 10.1099/00221287-76-1-177 4723067

[aps311335-bib-0016] Kinyua, Z. M. , S. A. Miller , C. Ashlina , and S. Nagendra . 2014 Bacterial wilt disease caused by *Ralstonia solanacearum*: Standard operating procedure for use in diagnostic laboratories. Version: EA‐SOP‐RS1, May 2014. Website https://ipmil.cired.vt.edu/wp-content/uploads/2014/06/SOP-Ralstonia-solanacerum-EastAfricaFinal-Apr2014-2.pdf [accessed 9 March 2020].

[aps311335-bib-0017] Kumar, R. , A. Barman , G. Jha , and S. Ray . 2013 Identification and establishment of genomic identity of *Ralstonia solanacearum* isolated from a wilted chilli plant at Tezpur, North East India. Current Science 105: 1571–1578.

[aps311335-bib-0018] Liu, H. , Y. Kang , S. Genin , M. A. Schell , and T. P. Denny . 2001 Twitching motility of *Ralstonia solanacearum* requires a type IV pilus system. Microbiology 147: 3215–3229. 11739754 10.1099/00221287-147-12-3215

[aps311335-bib-0019] Lowe‐Power, T. M. , C. G. Hendrich , E. Von Roepenack‐Lahaye , B. Li , D. Wu , R. Mitra , B. L. Dalsing , et al. 2018 Metabolomics of tomato xylem sap during bacterial wilt reveals *Ralstonia solanacearum* produces abundant putrescine, a metabolite that accelerates wilt disease. Environmental Microbiology 20: 1330–1349. 29215193 10.1111/1462-2920.14020 PMC5903990

[aps311335-bib-0020] Minh Tran, T. , A. Macintyre , D. Khokhani , M. Hawes , and C. Allen . 2016 Extracellular DNases of *Ralstonia solanacearum* modulate biofilms and facilitate bacterial wilt virulence. Environmental Microbiology 18: 4103–4117. 27387368 10.1111/1462-2920.13446

[aps311335-bib-0021] Monteiro, F. , M. Solé , I. V. Dijk , and M. Valls . 2012 A chromosomal insertion toolbox for promoter probing, mutant complementation, and pathogenicity studies in *Ralstonia solanacearum* . Molecular Plant‐Microbe Interactions 25: 557–568. 22122329 10.1094/MPMI-07-11-0201

[aps311335-bib-0022] Netting, A. G. , J. C. Theobald , and I. C. Dodd . 2012 Xylem sap collection and extraction methodologies to determine in vivo concentrations of ABA and its bound forms by gas chromatography‐mass spectrometry (GC‐MS). Plant Methods 8: 11. 22439865 10.1186/1746-4811-8-11 PMC3337791

[aps311335-bib-0023] OEPP/EPPO . 2004 Diagnostic protocols for regulated pests: *Ralstonia solanacearum* . EPPO Bulletin 34: 173–178.

[aps311335-bib-0024] Peyraud, R. , L. Cottret , L. Marmiesse , J. Gouzy , and S. Genin . 2016 A resource allocation trade‐off between virulence and proliferation drives metabolic versatility in the plant pathogen *Ralstonia solanacearum* . PLoS Pathogens 12: e1005939. 27732672 10.1371/journal.ppat.1005939 PMC5061431

[aps311335-bib-0025] Ramegowda, V. , M. Senthil‐Kumar , Y. Ishiga , A. Kaundal , M. Udayakumar , and K. S. Mysore . 2013 Drought stress acclimation imparts tolerance to *Sclerotinia sclerotiorum* and *Pseudomonas syringae* in *Nicotiana benthamiana* . International Journal of Molecular Sciences 14: 9497–9513. 23644883 10.3390/ijms14059497 PMC3676796

[aps311335-bib-0026] Rellán‐Álvarez, R. , H. El‐Jendoubi , G. Wohlgemuth , A. Abadía , O. Fiehn , J. Abadía , and A. Álvarez‐Fernández . 2011 Metabolite profile changes in xylem sap and leaf extracts of Strategy I plants in response to iron deficiency and resupply. Frontiers in Plant Science 2: 66. 22645546 10.3389/fpls.2011.00066 PMC3355808

[aps311335-bib-0027] Scholander, P. F. , E. D. Bradstreet , E. A. Hemmingsen , and H. T. Hammel . 1965 Sap pressure in vascular plants: Negative hydrostatic pressure can be measured in plants. Science 148: 339–346. 17832103 10.1126/science.148.3668.339

[aps311335-bib-0028] Singh, N. , T. Phukan , P. L. Sharma , K. Kabyashree , A. Barman , R. Kumar , R. V. Sonti , et al. 2018 An innovative root inoculation method to study *Ralstonia solanacearum* pathogenicity in tomato seedlings. Phytopathology 108: 436–442. 29182472 10.1094/PHYTO-08-17-0291-R

[aps311335-bib-0029] Tans‐Kersten, J. , H. Huang , and C. Allen . 2001 *Ralstonia solanacearum* needs motility for invasive virulence on tomato. Journal of Bacteriology 183: 3597–3605. 11371523 10.1128/JB.183.12.3597-3605.2001 PMC95236

[aps311335-bib-0030] Yuliar, Y. , A. Nion , and K. Toyota . 2015 Recent trends in control methods for bacterial wilt diseases caused by *Ralstonia solanacearum* . Microbes and Environments 30: 1–11. 25762345 10.1264/jsme2.ME14144 PMC4356456

[aps311335-bib-0031] Zheng, X. , Y. Zhu , B. Liu , N. Lin , and D. Zheng . 2017 Invasive properties of *Ralstonia solanacearum* virulent and avirulent strains in tomato roots. Microbial Pathogenesis 113: 144–151. 29074427 10.1016/j.micpath.2017.10.046

